# Supplementary material for: Linear and nonlinear correlation estimators unveil undescribed taxa interactions in microbiome data
Source: Nat Commun. 2022 Aug 23;13:4946. doi: 10.1038/s41467-022-32243-x (PMC9399263; doi:10.1038/s41467-022-32243-x)
Supplement: Supplementary file 1 — Supplementary Information [file 41467_2022_32243_MOESM1_ESM.pdf]

Supplementary Information

Linear and nonlinear correlation estimators unveil undescribed taxa interactions in microbiome data

Huang Lin[1], Merete Eggesbø[2], and Shyamal Das Peddada[1,*]

[1]Biostatistics and Bioinformatics Branch, NICHD, NIH, Bethesda, MD, USA
[2]Norwegian Institute of Public Health, Oslo, Norway
[*]shyamal.peddada@nih.gov

Supplementary Methods

Simulation details

Linear correlations. We generated $n = 50$ independent samples with $d = 100$ taxa, $n = 100$ independent samples with $d = 200$ taxa from Negative-Binomial distributions $A \sim NB(\mu, \alpha)$, where $\mu = 2000$ represents rare taxa, $\mu = 10,000$ represents low abundant taxa, $\mu = 40,000$ represents medium abundant taxa, and $\mu = 100,000$ represents high abundant taxa. The proportions of rare, low, median, and high abundant taxa were set to be 10%, 40%, 40%, 10%. The dispersion parameter $\alpha = 0.5$ or 2, representing low and high over dispersion, respectively. For the first 50 taxa, each taxon was set to be linearly correlated with its adjacent taxon. Specifically, the underlying true correlation coefficient matrix was:

$$\mathbf{R}^0 = [\rho^0_{lm}]_{l,m=1,\dots,d} = \begin{bmatrix} \mathbf{B}_{50\times50} & \mathbf{0}_{(d-50)\times50} \\ \mathbf{0}_{(d-50)\times50} & \mathbf{I}_{(d-50)\times(d-50)} \end{bmatrix},$$

where

(1) $\mathbf{B} = \mathbf{I}_{25\times25} \otimes \mathbf{1}\mathbf{1}^T, \mathbf{1} = (1,1)^T, \otimes$ is the Kronecker product,

(2) $\mathbf{0}$ is the zero matrix,

(3) $\mathbf{I}$ is the identity matrix.

The iteration number for each (n, d, α) combination was set to 100, and the significance level was set to be 0.001. To obtain sparse correlation matrices for other competing methods, the following strategies were implemented for each method. Standard Pearson correlation coefficient: used the same p-value filtering strategy as in SECOM (Pearson2) and SECOM (Distance). SparCC: Used a cutoff of $\rho_t = 0.3$ as recommended in Friedman et al. [1], i.e., $\hat{\rho}_{lm} = \hat{\rho}_{lm} I(|\hat{\rho}_{lm}| > \rho_t)$. Proportionality method: To deal with the asymmetry issue, the symmetrized form $\phi_{lm} = \min(\phi_{lm}, \phi_{ml})$ was used. Since the closer ϕ is to zero, the stronger the proportionality, we set the 5th percentile of all ϕ_{lm} (denoted it as ϕ_5) as the cutoff value, i.e., $\phi_{lm} = \phi_{lm} I(\phi_{lm} \leq \phi_5)$. SPIEC-EASI: We used a larger number of stars repetitions ($rep.num = 50$), and set the number of penalties ($nlambda$) to be 20, the min/max ratio of the penalty parameter ($lambda.min.ratio$) to be 0.01 as recommended in the corresponding R package user manual.

Nonlinear correlations. We generated $n = 50$ independent samples with $d = 100$ taxa, $n = 100$ independent samples with $d = 200$ taxa from Log-Normal distributions $A \sim LN(\delta, \sigma^2)$ (so $a = \log A \sim (\delta, \sigma^2)$), where $\delta = \log\mu - \frac{1}{2}\sigma^2$ and $\sigma^2 = log(\frac{\mu+\alpha\mu^2}{\mu^2} + 1)$. The Log-Normal distribution was selected for its convenience in specifying the covariance structure, and the parameters of Log-Normal distribution $A \sim LN(\delta, \sigma^2)$ were specified so that $\mathbb{E}(A)$ and $Var(A)$ were the same as those from Negative-Binomial distributions $A \sim NB(\mu, \alpha)$. As stated above, $\mu = 2000$ represents rare taxa, $\mu = 10,000$ represents low abundant taxa, $\mu = 40,000$ represents medium abundant taxa, and $\mu = 100,000$ represents high abundant taxa. The proportions of rare, low, median, and high abundant taxa were set to be 10%, 40%, 40%, 10%. The dispersion parameter $\alpha = 0.5$ or 2, representing low and high over dispersion, respectively. For the first 50 taxa, each taxon was set to be quadratically correlated with its adjacent taxon. Specifically, the following pairs, $(a_{1j}, a_{2j}), (a_{3j}, a_{4j}), \dots, (a_{47,j}, a_{49,j}), (a_{49,j}, a_{50,j})$, were quadratically correlated. The rest of taxa were uncorrelated. The iteration number for each (n, d, α) combination was set to 100, and the significance level was set to be 0.001. Strategies to obtain sparse correlation matrices were the same as those implemented in simulations of linear correlations.

Multiple ecosystems. Two absolute abundance tables (ecosystems) were generated from Negative-Binomial distributions $A \sim NB(\mu, \alpha)$, where the sample size and the number of taxa were $(n = 50, d = 100)$ or $(n = 100, d = 200)$ as above. Similarly, $\mu = 2000$ represents rare taxa, $\mu = 10,000$ represents low abundant taxa, $\mu = 40,000$ represents medium abundant taxa, and $\mu = 100,000$ represents high abundant taxa. The proportions of rare, low, median, and high abundant taxa were set to be 10%, 40%, 40%, 10%. The dispersion parameter $\alpha = 0.5$ or 2, representing low and high over dispersion, respectively. For the first 50 taxa in ecosystem 1, each taxon was set to be linearly correlated with its corresponding taxon in ecosystem 2. The rest of taxa were uncorrelated either within or between ecosystems. Specifically, the underlying true correlation coefficients were:

$$\rho^0_{jj'} = 1I_{\{j=j' \text{ from the same ecosystem}\}} + 1I_{\{j=j' \in \{1,\ldots,50\} \text{ from different ecosystems}\}}. \tag{1}$$

The iteration number for each (n, d, α) combination was set to 100, and the significance level was set to be 0.001. Strategies to obtain sparse correlation matrices were the same as those implemented in simulations of linear correlations.

Comparison between CCA and Add One. 20 absolute abundances for a pair of taxa (A_1, A_2) were generated from Negative-Binomial distributions. For uncorrelated case, A_1, A_2 were generated independently from $NB(200, 2)$; while for the correlated case, $A_1 \sim NB(200, 2)$ and $A_2 = 2A_1$. Structural zeros were generated by forcing the the first 8 samples of A_1 and the last 8 samples of A_2 to be 0s, and set $O_1 = 0.1A_1$, $O_2 = 0.1A_2$. Sampling zeros were generated by introducing relatively small sampling fractions, i.e., $O_1 = 0.01A_1$ and $O_2 = 0.01A_2$.

Comparison between CCA and imputation methods (GBM and MICE). We generated $n = 100$ independent samples for $d = 50$ taxa from Log-Normal distributions $A \sim LN(\delta, \sigma^2)$ (so $a = \log A \sim (\delta, \sigma^2)$), where $\delta = \log \mu - \frac{1}{2}\sigma^2$ and $\sigma^2 = log(\frac{\mu+\alpha\mu^2}{\mu^2} + 1)$. The Log-Normal distribution was selected for its convenience in specifying the covariance structure, and the parameters of Log-Normal distribution $A \sim LN(\delta, \sigma^2)$ were specified so that $\mathbb{E}(A)$ and $Var(A)$ were the same as those from Negative-Binomial distributions $A \sim NB(\mu, \alpha)$. We set $\mu = 200, 1,000, 10,000$ and $\alpha = 2$, and focused on only one pair of taxa with $\mu = 200$ (the remaining 48 taxa were not surveyed but treated as predictors for GBM). For the uncorrelated case, the correlation coefficient was set to 0; while for the correlated case, the correlation coefficient was set to 0.8. A range of sampling fraction (0.1, 0.05, 0.02, 0.01, 0.005) was applied to the true abundances to generate (sampling) zeros, which corresponded to the proportion of zeros ranging from 0 to around 70%. The iteration number was set to 100.

Proofs of Theorems

Proofs presented below use some of the techniques developed in Kaul et al. (2017). [2].

Lemma 1: Suppose $a_{ij} - E(a_{ij})|M_{ij} = 1$ and e_{ij} follow sub-Gaussian distributions. For $1 \le i \le n, 1 \le j \le d$, denote

$$\mu_j = E(y_{ij}) = 0, \quad \sigma_{lm} = Cov(y_{il}, y_{im}), \quad \rho_{lm} = Corr(y_{il}, y_{im}), \quad R = [\rho_{lm}]_{l,m=1,\dots,d}.$$

Let $\eta_{ij} = \frac{y_{ij}-\mu_j}{\sqrt{\sigma_{jj}}}$, then with probability at least $1 - \exp(-c_1 \log d)$,

$$\max_{1\le l,m\le d} \frac{1}{|n(l,m)|}\Big| \sum_{i\in n(l,m)} (\eta_{il}\eta_{im} - \rho_{lm})\Big| \le c_0\sqrt{\frac{\log d}{n}},$$

for some finite positive constants c_0 and c_1.

Proof: First, since

$$y_{ij} = (a_{ij} - \bar{a}_{\cdot j}) + (e_{ij} - \bar{e}_{\cdot j}),$$

therefore

$$\eta_{ij} = \frac{y_{ij} - \mu_j}{\sqrt{\sigma_{jj}}} = \frac{\frac{n-1}{n}(a_{ij} - E(a_{ij})) - \frac{1}{n}\sum_{l\neq j}(a_{il} - E(a_{il})) + \frac{n-1}{n}e_{ij} - \frac{1}{n}\sum_{l\neq j} e_{il}}{\sqrt{\sigma_{jj}}},$$

which is the summation of independent sub-Gaussian random variables. Thus, η_{ij} also follows a sub-Gaussian distribution.

Observe that

$$\begin{aligned}\Big| \sum_{i\in n(l,m)} (\eta_{il}\eta_{im} - E\eta_{il}\eta_{im})\Big| &\le \frac{1}{4}\Big| \sum_{i\in n(l,m)} \Big[(\eta_{il} + \eta_{im})^2 - E(\eta_{il} + \eta_{im})^2\Big]\Big| \\ &+ \frac{1}{4}\Big| \sum_{i\in n(l,m)} \Big[(\eta_{il} - \eta_{im})^2 - E(\eta_{il} - \eta_{im})^2\Big]\Big| \\ &= (T1) + (T2). \qquad (2)\end{aligned}$$

For (T1), since the sum of sub-Gaussian random variables (not necessarily independent) is also sub-Gaussian, and the square of a sub-Gaussian random variable is sub-exponential [3], we have

$$(\eta_{il} + \eta_{im})^2 - E(\eta_{il} + \eta_{im})^2 \sim \text{subE}(\lambda), \quad \text{for some constant } \lambda > 0.$$

Therefore, by Bernstein's inequality,

$$\begin{aligned}P\Big(\frac{1}{|n(l,m)|} \sum_{i\in n(l,m)} \Big[(\eta_{il} + \eta_{im})^2 - E(\eta_{il} + \eta_{im})^2\Big] > t|M_i\Big) &\le \exp\Big[-\frac{|n(l,m)|}{2}\min(\frac{t^2}{\lambda^2}, \frac{t}{\lambda})\Big] \\ &\le \exp(-\frac{|n(l,m)|t^2}{2\lambda^2}).\end{aligned}$$

Applying a union bound and monotonic property of conditional expectation, we have

$$P\Big(\max_{1\le l,m\le d} \frac{1}{|n(l,m)|}\Big| \sum_{i\in n(l,m)} \Big[(\eta_{il}+\eta_{im})^2 - E(\eta_{il}+\eta_{im})^2\Big]\Big| > t\Big) \le 2d^2 \max_{1\le l,m\le d} E\exp(-\frac{|n(l,m)|t^2}{2\lambda^2}). \qquad (3)$$

On the other hand, note that $|n(l,m)| = \sum_{i=1}^n I_i(l,m)$, where, $I_i(l,m) = I(M_{il} = M_{im} = 1)$ for any $1 \le l, m \le d$. Since $I_i(l,m)$ are independent r.v.'s over $1 \le i \le n$, we have

$$
\begin{aligned}
\max_{1\le l,m\le d} E\exp(-\frac{|n(l,m)|t^2}{2\lambda^2}) &= \max_{1\le l,m\le d} \exp\Big\{-\frac{t^2}{2\lambda^2}E|n(l,m)|\Big\}E\exp\Big\{-\frac{t^2}{2\lambda^2}(|n(l,m)| - E|n(l,m)|)\Big\} \\
&= \max_{1\le l,m\le d} \exp\Big\{-\frac{t^2}{2\lambda^2}\sum_{i=1}^n \delta(l,m)\Big\}E\exp\Big\{-\frac{t^2}{2\lambda^2}(|n(l,m)| - E|n(l,m)|)\Big\} \\
&\le \exp(-\frac{nt^2\delta_{min}}{2\lambda^2}) \max_{1\le l,m\le d} E\exp\Big\{-\frac{t^2}{2\lambda^2}(|n(l,m)| - E|n(l,m)|)\Big\} \\
&= \exp(-\frac{nt^2\delta_{min}}{2\lambda^2}) \max_{1\le l,m\le d} (T3). \qquad (4)
\end{aligned}
$$

Since $|I_i(l,m) - EI_i(l,m)| \le 1$, and $|n(l,m)| - E|n(l,m)| = \sum_{i=1}^n \Big[I_i(l,m) - EI_i(l,m)\Big]$, $|n(l,m)| - E|n(l,m)| \sim \text{subG}(n)$, therefore by Hoeffding's inequality,

$$
(T3) \le \exp(\frac{\frac{t^4}{4\lambda^4}\cdot n}{2}) = \exp(\frac{nt^4}{8\lambda^4}). \qquad (5)
$$

Based on (3), (4), and (5), we obtain the upper bound for (T1) as

$$
P\Big(\max_{1\le l,m\le d} \frac{1}{|n(l,m)|}\Big|\sum_{i\in n(l,m)} \Big[(\eta_{il}+\eta_{im})^2 - E(\eta_{il}+\eta_{im})^2\Big]\Big| > t\Big) \le 2d^2\exp(-\frac{nt^2\delta_{min}}{2\lambda^2})\exp(\frac{nt^4}{8\lambda^4}).
$$

Similar to (T1), for (T2), we have

$$
P\Big(\max_{1\le l,m\le d} \frac{1}{|n(l,m)|}\Big|\sum_{i\in n(l,m)} \Big[(\eta_{il}-\eta_{im})^2 - E(\eta_{il}-\eta_{im})^2\Big]\Big| > t\Big) \le 2d^2\exp(-\frac{nt^2\delta_{min}}{2\lambda^2})\exp(\frac{nt^4}{8\lambda^4}).
$$

Combining the upper bounds for (T1) and (T2), and plug into (2), we obtain

$$
P\Big(\max_{1\le l,m\le d} \frac{1}{|n(l,m)|}\Big|\sum_{i\in n(l,m)} (\eta_{il}\eta_{im} - E\eta_{il}\eta_{im})\Big| > t\Big) \le d^2\exp(-\frac{nt^2\delta_{min}}{2\lambda^2} + \frac{nt^4}{8\lambda^4}). \qquad (6)
$$

Choose a sufficiently small t such that

$$
-\frac{nt^2\delta_{min}}{2\lambda^2} + \frac{nt^4}{8\lambda^4} \le -\frac{nt^2\delta_{min}}{4\lambda^2}.
$$

Therefore,

$$
(6) \le d^2\exp(-\frac{nt^2\delta_{min}}{4\lambda^2}).
$$

Choosing $t = c_0\sqrt{\frac{\log d}{n}}$ and plugging into the above expression, we obtain

$$
(6) \le d^2\exp(-\frac{c_0^2\delta_{min}}{4\lambda^2}\log d) = \exp\Big\{-(\frac{c_0^2\delta_{min}}{4\lambda^2} - 2)\log d\Big\}.
$$

Choose c_0 such that

$$
\frac{c_0^2\delta_{min}}{4\lambda^2} - 2 > 0,
$$

we then obtain the right hand side of (6) $\le \exp(-c_1\log d)$. This completes the proof.

Lemma 2: Suppose the assumptions of Lemma 1 hold. Let

$$\tilde{\mu}_j = \frac{1}{|n(j)|}\sum_{i\in n(j)} y_{ij}, \quad \tilde{\sigma}_{lm} = \frac{1}{|n(l,m)|}\sum_{i\in n(l,m)} (y_{il}-\tilde{\mu}_l)(y_{im}-\tilde{\mu}_m),$$
$$\tilde{\rho}_{lm} = \frac{\tilde{\sigma}_{lm}}{\sqrt{\tilde{\sigma}_{ll}\tilde{\sigma}_{mm}}}, \quad \tilde{R} = [\tilde{\rho}_{lm}]_{l,m=1,\dots,d}.$$

Then, for some positive finite constants c_1 and, c_2, $\|\tilde{R}-R\|_\infty \le c_2\sqrt{\frac{\log d}{n}}$, with a probability of at least $1-\exp(-c_1\log d)$.

Proof: Let $\tilde{\eta}_{ij} = \frac{y_{ij}-\tilde{\mu}_j}{\sqrt{\tilde{\sigma}_{jj}}}$, we want to prove that

$$\max_{1\le l,m\le d}\frac{1}{|n(l,m)|}\Big|\sum_{i\in n(l,m)}(\tilde{\eta}_{il}\tilde{\eta}_{im}-\rho_{lm})\Big| \le c_2\sqrt{\frac{\log d}{n}}, \tag{7}$$

with probability at least $1-\exp(-c_1\log d)$.

Define the event

$$\mathbb{A} = \Big\{\max_{1\le l,m\le d}|\tilde{\sigma}_{lm}-\sigma_{lm}| \le c_0K^2\sqrt{\frac{\log d}{n}}\Big\},$$

where K is the upper bound for σ_{jj} defined in Assumption 0.1. Then from Lemma 1 we have

$$P(\mathbb{A}) \ge 1-\exp(-c_1\log d).$$

Note that

$$\begin{aligned}
|\tilde{\rho}_{lm}-\rho_{lm}| &= \Big|\frac{\tilde{\sigma}_{lm}}{\sqrt{\tilde{\sigma}_{ll}\tilde{\sigma}_{mm}}} - \frac{\sigma_{lm}}{\sqrt{\sigma_{ll}\sigma_{mm}}}\Big| \\
&= \Big|\frac{\tilde{\sigma}_{lm}}{\sqrt{\tilde{\sigma}_{ll}\tilde{\sigma}_{mm}}} - \frac{\tilde{\sigma}_{lm}}{\sqrt{\sigma_{ll}\sigma_{mm}}} + \frac{\tilde{\sigma}_{lm}}{\sqrt{\sigma_{ll}\sigma_{mm}}} - \frac{\sigma_{lm}}{\sqrt{\sigma_{ll}\sigma_{mm}}}\Big| \\
&\le \Big|\frac{\tilde{\sigma}_{lm}}{\sqrt{\tilde{\sigma}_{ll}\tilde{\sigma}_{mm}}} - \frac{\tilde{\sigma}_{lm}}{\sqrt{\sigma_{ll}\sigma_{mm}}}\Big| + \Big|\frac{\tilde{\sigma}_{lm}}{\sqrt{\sigma_{ll}\sigma_{mm}}} - \frac{\sigma_{lm}}{\sqrt{\sigma_{ll}\sigma_{mm}}}\Big| \\
&= |A_{lm}| + |B_{lm}|.
\end{aligned} \tag{8}$$

Within event $\mathbb{A}$, it is easy to verify that

$$\max_{1\le l,m\le d}|B_{lm}| \le c_0\sqrt{\frac{\log d}{n}}. \tag{9}$$

On the other hand, $A_{ij} = \frac{\tilde{\sigma}_{lm}}{\sqrt{\tilde{\sigma}_{ll}\tilde{\sigma}_{mm}}}\Big(1-\sqrt{\frac{\tilde{\sigma}_{ll}\tilde{\sigma}_{mm}}{\sigma_{ll}\sigma_{mm}}}\Big)$. Therefore $|A_{ij}| \le 1+\sqrt{\frac{\tilde{\sigma}_{ll}\tilde{\sigma}_{mm}}{\sigma_{ll}\sigma_{mm}}}$. Also, within event $\mathbb{A}$,

$$\max_{1\le l,m\le d}\frac{\sigma_{lm}}{\tilde{\sigma}_{lm}} \le 1+\frac{c_0K^2}{\tilde{\sigma}_{lm}}\sqrt{\frac{\log d}{n}} \le c_3\sqrt{\frac{\log d}{n}}.$$

Thus, if event $\mathbb{A}$ holds,

$$\max_{1\le l,m\le d}|A_{lm}| \le c_4\sqrt{\frac{\log d}{n}}. \tag{10}$$

Combining (8), (9), and (10), we therefore have $\max_{1\le l,m\le d}|\tilde{\rho}_{lm}-\rho_{lm}| \le c_2\sqrt{\frac{\log d}{n}}$ with probability at least $1-\exp(-c_1\log d)$.

Lemma 3: If Assumption 0.1 holds, then with probability at least $1 - c_3 \frac{n}{d^{c_5} \log d}$

$$\left|\widehat{s_i - \bar{s}.} - (s_i - \bar{s}.)\right| \le c_4 \sqrt{\frac{\log d}{n}},$$

for some constant $0 < c_5 \le 1, 0 < c_3, c_4 < \infty$, where

$$\widehat{s_i - \bar{s}.} = \sum_{j \in d(i)} w_j (o_{ij} - \bar{o}_{\cdot j}), \quad w_j = \frac{1}{Var(o_{ij} - \bar{o}_{\cdot j})} / \sum_{l \in d(i)} \frac{1}{Var(o_{il} - \bar{o}_{\cdot l})}.$$

Proof: First of all, note that

$$o_{ij} - \bar{o}_{\cdot j} = (s_i - \bar{s}.) + (a_{ij} - \bar{a}_{\cdot j}) + (e_{ij} - \bar{e}_{\cdot j}),$$

therefore

$$\begin{aligned} Var(\widehat{s_i - \bar{s}.}) &= w_j^2 \sum_{j \in d(i)} Var(o_{ij} - \bar{o}_{\cdot j}) + \sum_{l \neq m} w_l w_m Cov(o_{il} - \bar{o}_{\cdot l}, o_{im} - \bar{o}_{\cdot m}), \\ &= O(d^{-2}) O(d) + \sum_{l \neq m} w_l w_m Cov(a_{il} - \bar{a}_{\cdot l}, a_{im} - \bar{a}_{\cdot m}), \\ &= O(d^{-1}) + O(d^{-2}) \sum_{l \neq m} (1 - \frac{1}{n}) \sigma_{lm}^0, \\ &= O(d^{-1}) + O(d^{-2}) o(d^2), \quad \text{based on Assumption 0.1}, \\ &= O(d^{-1}) + o(1), \\ &= O(d^{-c_5}), \end{aligned}$$

for some $0 < c_5 \le 1$. Also, since $E(\widehat{s_i - \bar{s}.}) = s_i - \bar{s}.$, by Chebyshev's inequality, we have

$$P\Big(\left|\widehat{s_i - \bar{s}.} - (s_i - \bar{s}.)\right| \ge t\Big) \le \frac{Var(\widehat{s_i - \bar{s}.})}{t^2}. \tag{11}$$

Choosing $t = c_3 \sqrt{\frac{\log d}{n}}$ in (11), the right hand side of (11) reduces to

$$O(d^{-c_5}) O(\frac{n}{\log d}) = O(\frac{n}{d^{c_5} \log d}),$$

which completes the proof.

Proof of Theorem 0.1

Let

$$\begin{aligned} \hat{\mu}_j &= \frac{1}{|n(j)|} \sum_{i \in n(j)} \hat{y}_{ij}, \quad \hat{\sigma}_{lm} = \frac{1}{|n(l,m)|} \sum_{i \in n(l,m)} (\hat{y}_{il} - \hat{\mu}_l)(\hat{y}_{im} - \hat{\mu}_m), \\ \hat{\rho}_{lm} &= \frac{\hat{\sigma}_{lm}}{\sqrt{\hat{\sigma}_{ll} \hat{\sigma}_{mm}}}, \quad R_n = [\hat{\rho}_{lm}]_{l,m=1,\dots,d}. \end{aligned}$$

We want to prove that $\|R_n - R\|_\infty = O_p\Big(\sqrt{\frac{\log d}{n}}\Big)$.

First, observe that $\|R_n - R\|_\infty \le \|R_n - \tilde{R}\|_\infty + \|\tilde{R} - R\|_\infty$. From Lemma 2 we have $\|\tilde{R} - R\|_\infty = O_p\Big(\sqrt{\frac{\log d}{n}}\Big)$. What remains is to bound $\|R_n - \tilde{R}\|_\infty$.

Let $\hat{\eta}_{ij} = \frac{\hat{y}_{ij} - \hat{\mu}_j}{\sqrt{\hat{\sigma}_{jj}}}$ and event $\mathbb{B} = \Big\{\Big|\widehat{s_i - \bar{s}.} - (s_i - \bar{s}.)\Big| = O_p\Big(\sqrt{\frac{\log d}{n}}\Big)\Big\}$. Then, from Lemma 3 we have $P(\mathbb{B}) \ge 1 - c_3 \frac{n}{d^{c_5} \log d}$.

Since $y_{ij} - \hat{y}_{ij} = \widehat{s_i - \bar{s}.} - (s_i - \bar{s}.)$, therefore it is easy to verify that within the event $\mathbb{B}$, $\Big|\hat{y}_{ij} - y_{ij}\Big| = O_p\Big(\sqrt{\frac{\log d}{n}}\Big)$ and $\Big|\hat{y}_{ij} - \hat{\mu}_j - (y_{ij} - \tilde{\mu}_j)\Big| = O_p\Big(\sqrt{\frac{\log d}{n}}\Big)$. Define $\hat{y}_{ij} - \hat{\mu}_j = y_{ij} - \tilde{\mu}_j + \delta$, and $\tilde{\eta}_{ij} = \frac{y_{ij} - \tilde{\mu}_j}{\sqrt{\tilde{\sigma}_{jj}}} = \frac{c_1}{c_2}$, then

$$\hat{\eta}_{ij} = \frac{y_{ij} - \tilde{\mu}_j + \delta}{\sqrt{\tilde{\sigma}_{jj} + \frac{1}{n(j)} \sum_{i \in n(j)} (y_{ij} - \tilde{\mu}_j)\delta + \delta^2}} = \frac{c_1 + \delta}{\sqrt{c_2^2 + \delta^2}},$$

where $c_1, c_2 < \infty$. Since $\delta = o(1)$, therefore

$$\hat{\eta}_{ij} - \tilde{\eta}_{ij} = \frac{c_1 c_2 + \delta c_2 - c_1 \sqrt{c_2^2 + \delta^2}}{c_2 \sqrt{c_2^2 + \delta^2}} \approx \frac{c_1 c_2 + \delta c_2 - c_1 c_2}{c_2 \sqrt{c_2^2 + \delta^2}} = \frac{\delta}{\sqrt{c_2^2 + \delta^2}} = O(\delta).$$

Thus, on event $\mathbb{B}$,

$$|\hat{\eta}_{ij} - \tilde{\eta}_{ij}| = O_p\Big(\sqrt{\frac{\log d}{n}}\Big). \tag{12}$$

Since

$$\hat{\rho}_{lm} = \frac{1}{|n(l,m)|} \sum_{i \in n(l,m)} \hat{\eta}_{il} \hat{\eta}_{im},$$

based on (12), one can verify that on event $\mathbb{B}$,

$$\max_{1 \le l,m \le d} \frac{1}{|n(l,m)|} \Big| \sum_{i \in n(l,m)} (\hat{\eta}_{il} \hat{\eta}_{im} - \tilde{\eta}_{il} \tilde{\eta}_{im}) \Big| = O_p\Big(\sqrt{\frac{\log d}{n}}\Big),$$

which completes the proof.

Proof of Theorem 0.2

Start with defining

$$\zeta_{ij} = \frac{r_{ij} - E(r_{ij})}{\sqrt{Var(r_{ij})}}, 1 \le l \le d, 1 \le i \le n.$$

The proof is very similar to the proof of Theorem 0.1, except that we don't need to assume $a_{ij}|M_{ij} = 1$ and e_{ij} follow sub-Gaussian distributions, since given the configuration of taxa presence $M_i, i = 1, \ldots, n$, assuming no ties, note that

$$E(r_{ij}) = \frac{|n(j)| + 1}{2}$$
$$Var(r_{ij}) = E(r_{ij}^2) - [E(r_{ij})]^2 = \frac{(|n(j)| + 1)(|n(j)| - 1)}{12}.$$

Therefore, we have

$$\min_{j\in n(j)} \zeta_{ij} = \frac{1-E(r_{ij})}{\sqrt{Var(r_{ij})}} = -\frac{\sqrt{12}}{2}\sqrt{\frac{|n(j)|-1}{|n(j)|+1}} \geq -\frac{\sqrt{12}}{2}$$
$$\max_{j\in n(j)} \zeta_{ij} = \frac{n-E(r_{ij})}{\sqrt{Var(r_{ij})}} = \frac{\sqrt{12}}{2}\sqrt{\frac{|n(j)|-1}{|n(j)|+1}} \leq \frac{\sqrt{12}}{2},$$

which implies that

$$\zeta_{ij} \in [-\frac{\sqrt{12}}{2}, \frac{\sqrt{12}}{2}] \text{ with } E(\zeta_{ij}) = 0.$$

By Hoeffding's lemma, $\zeta_{ij} \sim subG(\sigma^2 = 3)$.

Proof of Theorem 0.3

Let

$$\hat{\Delta} = \hat{R} - R = \arg\min_{\Delta} F(\Delta) \quad \text{s.t. } R + \Delta \succeq \epsilon I, \Delta_{jj} = 0,$$

where $F(\Delta) = \frac{1}{2}\|R + \Delta - R_n\|_F^2 + \lambda|R + \Delta|_1$.

Based on Theorem 0.1, define the event

$$\mathbb{C} = \Big\{|\hat{\rho}_{lm} - \rho_{lm}| \leq \lambda\Big\},$$

where $\lambda = O_p\Big(\sqrt{\frac{\log d}{n}}\Big)$.

On event $\mathbb{C}$, consider any $\Delta \in \mathbb{D}$ for

$$\mathbb{D} = \Big\{\Delta : \Delta = \Delta^T, R + \Delta \succeq \epsilon I, \Delta_{jj} = 0, j = 1, \ldots, d, \|\Delta\|_F > 5q^{1/2}\lambda\Big\},$$

where q is the cardinality of the non-diagonal support of R, denoted as $A_0 = \{l, m : l \neq m, \rho_{lm} \neq 0\}$.

Denote Δ_{A_0} as an matrix such that $[\Delta_{A_0}]_{lm} = [\Delta]_{lm}$ for $(l, m) \in A_0$ and $[\Delta_{A_0}]_{lm} = 0$ for $(l, m) \notin A_0$. We see that

$$\begin{aligned}
F(\Delta) - F(0) &= \frac{1}{2}\|R + \Delta - R_n\|_F^2 - \frac{1}{2}\|R - R_n\|_F^2 + \lambda(|R + \Delta|_1 - |R|_1) \\
&= \frac{1}{2}\|\Delta\|_F^2 + \Big\langle \Delta, R - R_n \Big\rangle + \lambda|\Delta_{A_0^C}|_1 + \lambda(|R_{A_0} + \Delta_{A_0}|_1 - |R_{A_0}|_1) \\
&\geq \frac{1}{2}\|\Delta\|_F^2 - \lambda|\Delta|_1 + \lambda|\Delta_{A_0^C}|_1 - \lambda|\Delta_{A_0}|_1 \\
&= \frac{1}{2}\|\Delta\|_F^2 - 2\lambda|\Delta_{A_0}|_1 \\
&\geq \frac{1}{2}\|\Delta\|_F^2 - 2\lambda q^{1/2}\|\Delta\|_F \quad \text{by Cauchy-Schwartz ineq.} \\
&> 0 \quad \text{since } \Delta \in \mathbb{D}.
\end{aligned}$$

Because $G(\Delta) = F(\Delta) - F(0)$ is convex, $G(\Delta) > 0$ for any $\Delta \in \mathbb{D}$, and $G(0) = 0$, we see that the global optimal solution $\hat{\Delta}$ that minimizes $G(\Delta)$ must satisfy $\hat{\Delta} \notin \mathbb{D}$, i.e.

$$\|\hat{R} - R\|_F \leq 5q^{1/2}\lambda. \tag{13}$$

Therefore, on event $\mathbb{C}$, we have

$$\|\hat{R} - R\|_F^2 = O_p\Big(q\frac{\log d}{n}\Big).$$

Supplementary Figures

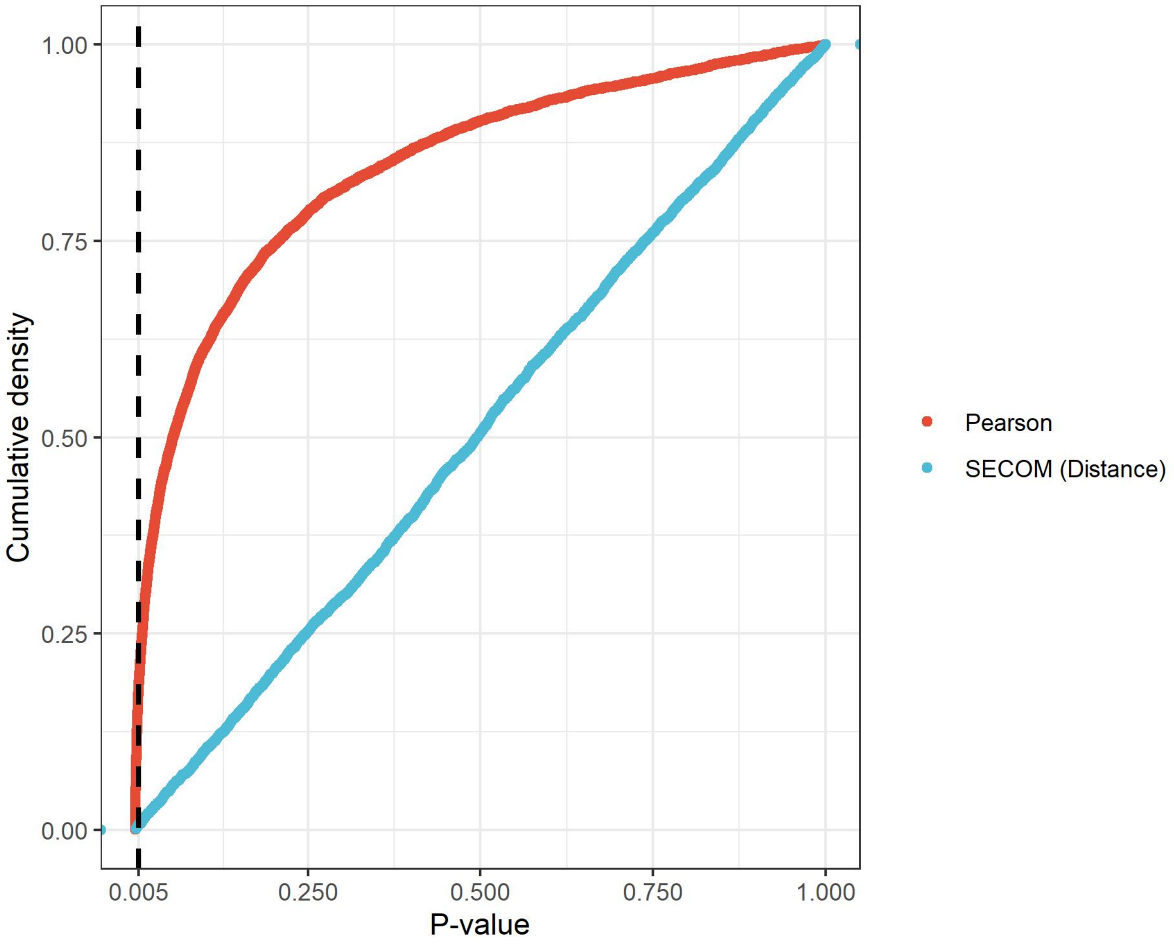

Supplementary Figure 1: The distribution of p-values (two-sided, not corrected for multiple comparisons) generated by standard Pearson correlation coefficient (red) and SECOM using distance correlation measure (blue) based on log observed abundances as shown in Fig. 1b. For the Pearson correlation coefficient, the p-values are derived using t-distribution. For the distance correlation, the p-value is calculated using a permutation test [4]. The vertical dashed line represents the p-value of 0.005. P-values generated by standard Pearson correlation coefficient do not account for differential sampling fractions and sequencing efficiencies. Consequently, larger the sequencing depth, the more the observed abundances. This results in inflated values for Pearson correlation coefficients, and thus an inflation of significant p-values. On the other hand, p-values generated by SECOM, which takes into account two sources of biases, do not suffer from inflation of false positives and thus, the distribution of p-values are approximately around the diagonal line representing the uniform distribution.

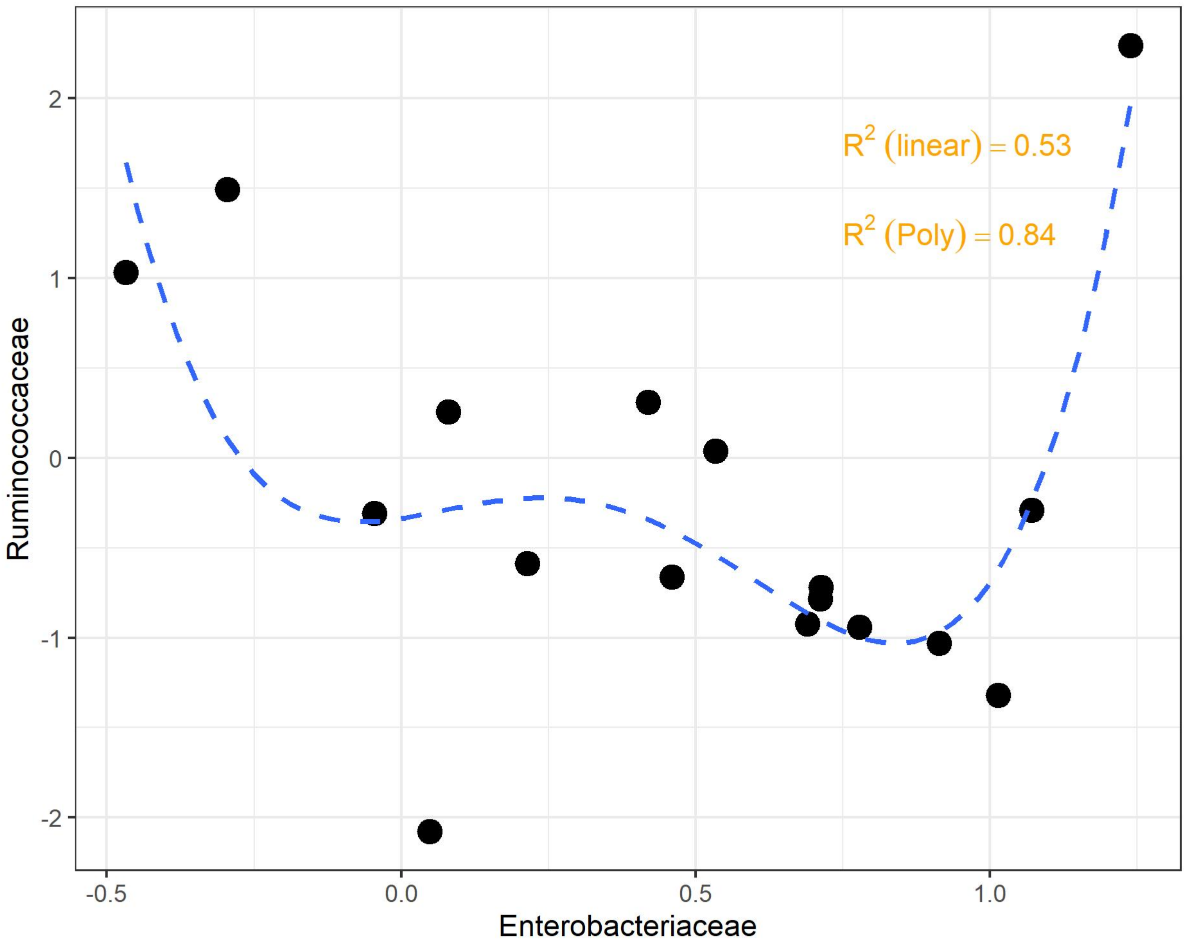

Supplementary Figure 2: The relationship between *Ruminococcaceae* and *Enterobacteriaceae* based on data obtained from the Norwegian Microbiota (NoMIC) study at day 120. The blue dashed line represents the robust polynomial regression (4th degree) fit. X- and y-axis represent the bias-corrected abundances for *Enterobacteriaceae* and *Ruminococcaceae*, respectively, derived from SECOM. The adjusted R^2 derived from MM-estimation using bisquare weighting for linear and polynomial regression (4th degree) are shown on the embedded text.

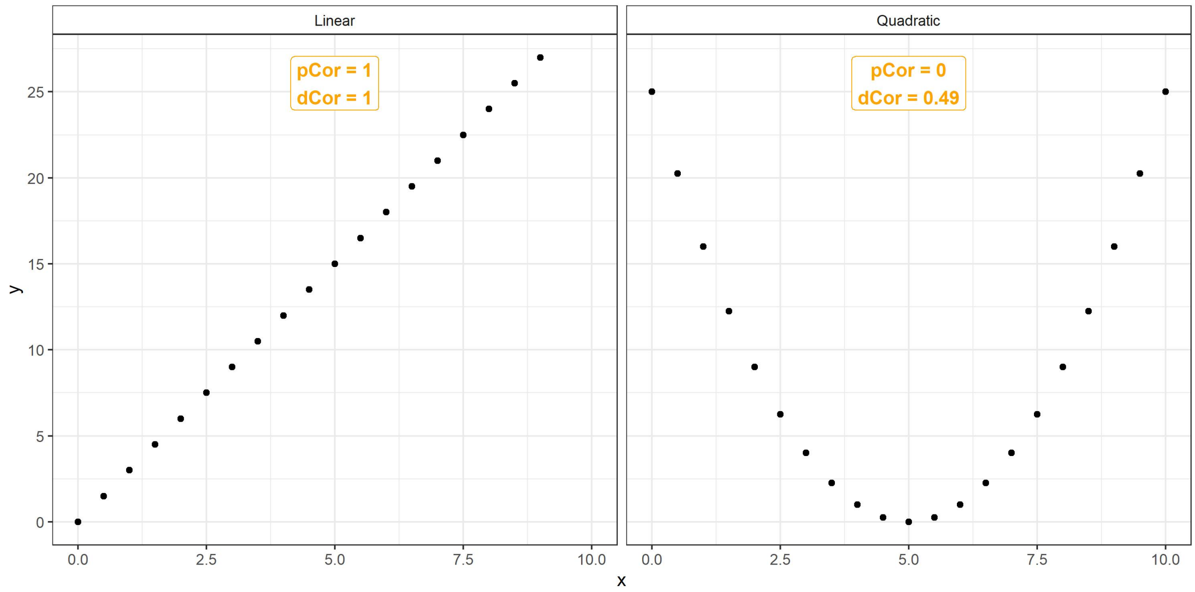

Supplementary Figure 3: Pearson correlation vs. Distance correlation.

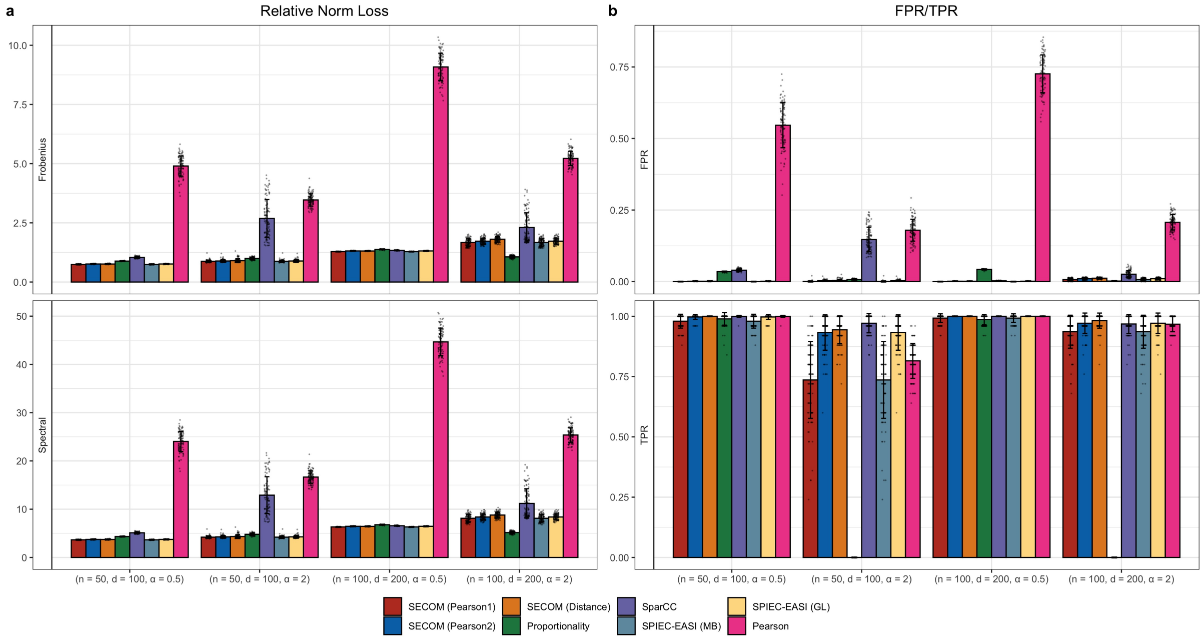

Supplementary Figure 4: Comparisons of estimation accuracy and false/true positive rate (FPR/TPR) for identifying linear relationships within an ecosystem. The average relative norm loss (Frobenius/Spectral) and FPR/TPR of various correlation methods (including the standard Pearson correlation coefficient) are shown in **a** and **b**, respectively. Synthetic data were generated from Negative-Binomial (NB) distributions. The X-axis denotes the simulation settings, which are a combination of sample size n, number of taxa d, and the dispersion parameter α. Results are represented by the average of corresponding measure (Frobenius/Spectral norm loss, or FPR/TPR) $\pm$ standard errors (shown as error bars) across 100 simulation runs for each $n/d/\alpha$ setting. Data points are added to the bar charts using dots with jittering. Color and the name of the corresponding correlation methods are shown at the bottom within the graph. The results demonstrate that SECOM and SPIEC-EASI outperformed all existing methods not only in terms of estimation accuracy, but also in terms of uniformly small FPR, and comparable TPR.

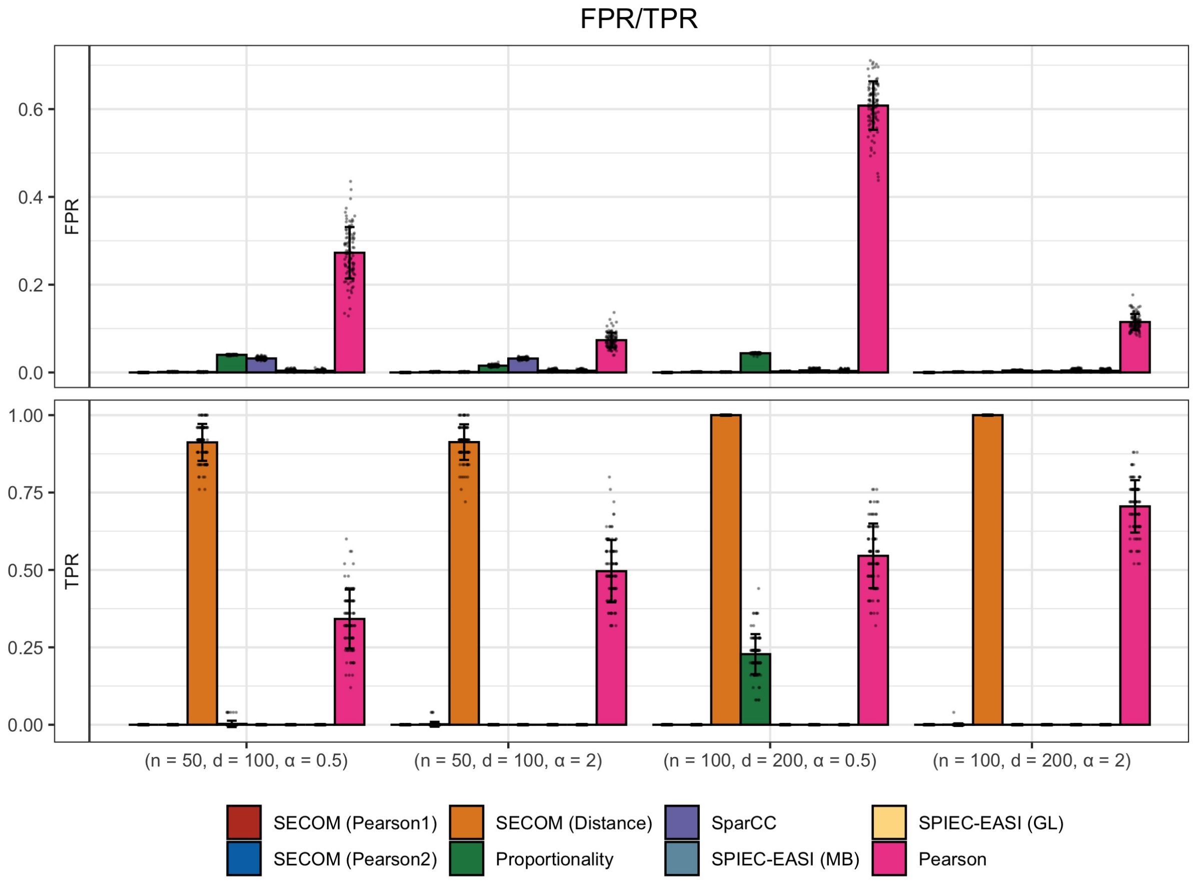

Supplementary Figure 5: Comparisons of false/true positive rate (FPR/TPR) for identifying nonlinear relationships (including the standard Pearson correlation coefficient) within an ecosystem. Synthetic data were generated from log-normal distributions. The X-axis denotes the simulation settings, which are a combination of sample size n, number of taxa d, and the dispersion parameter α. Results are represented by the average of corresponding measure (FPR/TPR) $\pm$ standard errors (shown as error bars) across 100 simulation runs for each $n/d/\alpha$ setting. Data points are added to the bar charts using dots with jittering. Color and the name of the corresponding correlation methods are shown at the bottom within the graph. Results showed that only SECOM controlled the FPR while maintaining high TPR. Other than SECOM, none of existing methods identify nonlinear relationships.

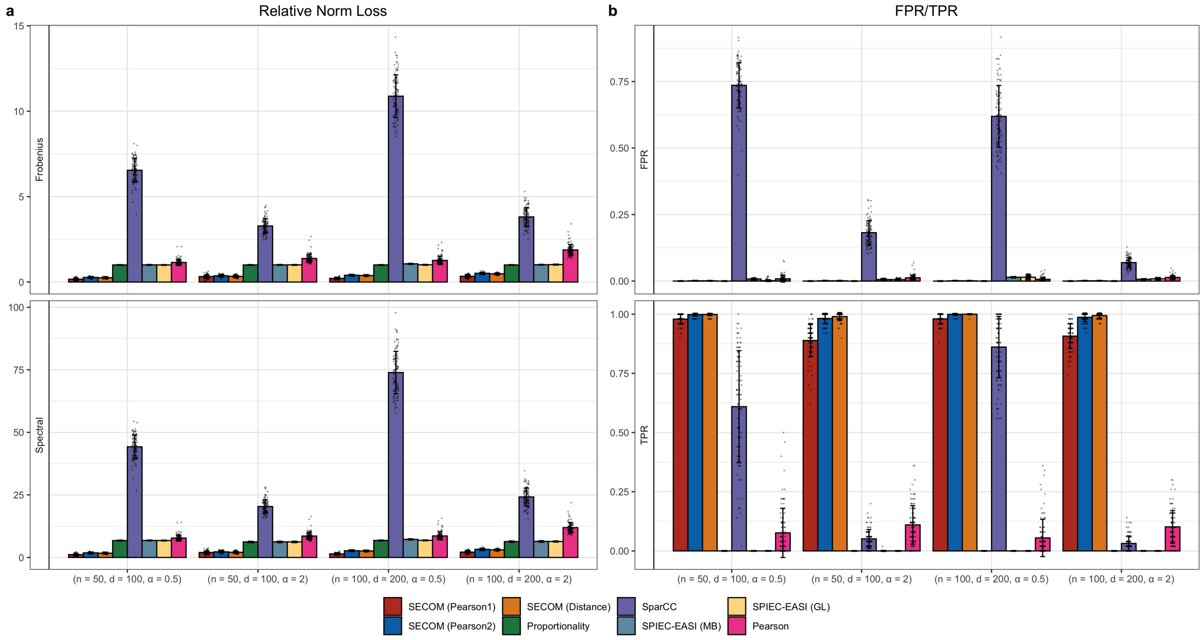

Supplementary Figure 6: Comparisons of estimation accuracy and false/true positive rate (FPR/TPR) for identifying linear relationships between two ecosystems. The relative norm loss (Frobenius/Spectral) and FPR/TPR of various correlation methods (including the standard Pearson correlation coefficient) are shown in **a** and **b**, respectively. Synthetic data were generated from Negative-Binomial (NB) distribution. The X-axis denotes the simulation settings, which are a combination of the sample size n, the number of taxa d, and the dispersion parameter α. Data from different ecosystems have different sampling fractions and sequencing efficiencies in each simulation run. Results are represented by the average of corresponding measure (Frobenius/Spectral norm loss, or FPR/TPR) $\pm$ standard errors (shown as error bars) across 100 simulation runs for each $n/d/\alpha$ setting. Data points are added to the bar charts using dots with jittering. Color and the name of the corresponding correlation methods are shown at the bottom within the graph. Results demonstrated that SECOM methods were the only methods to successfully quantify microbial correlations between ecosystems.

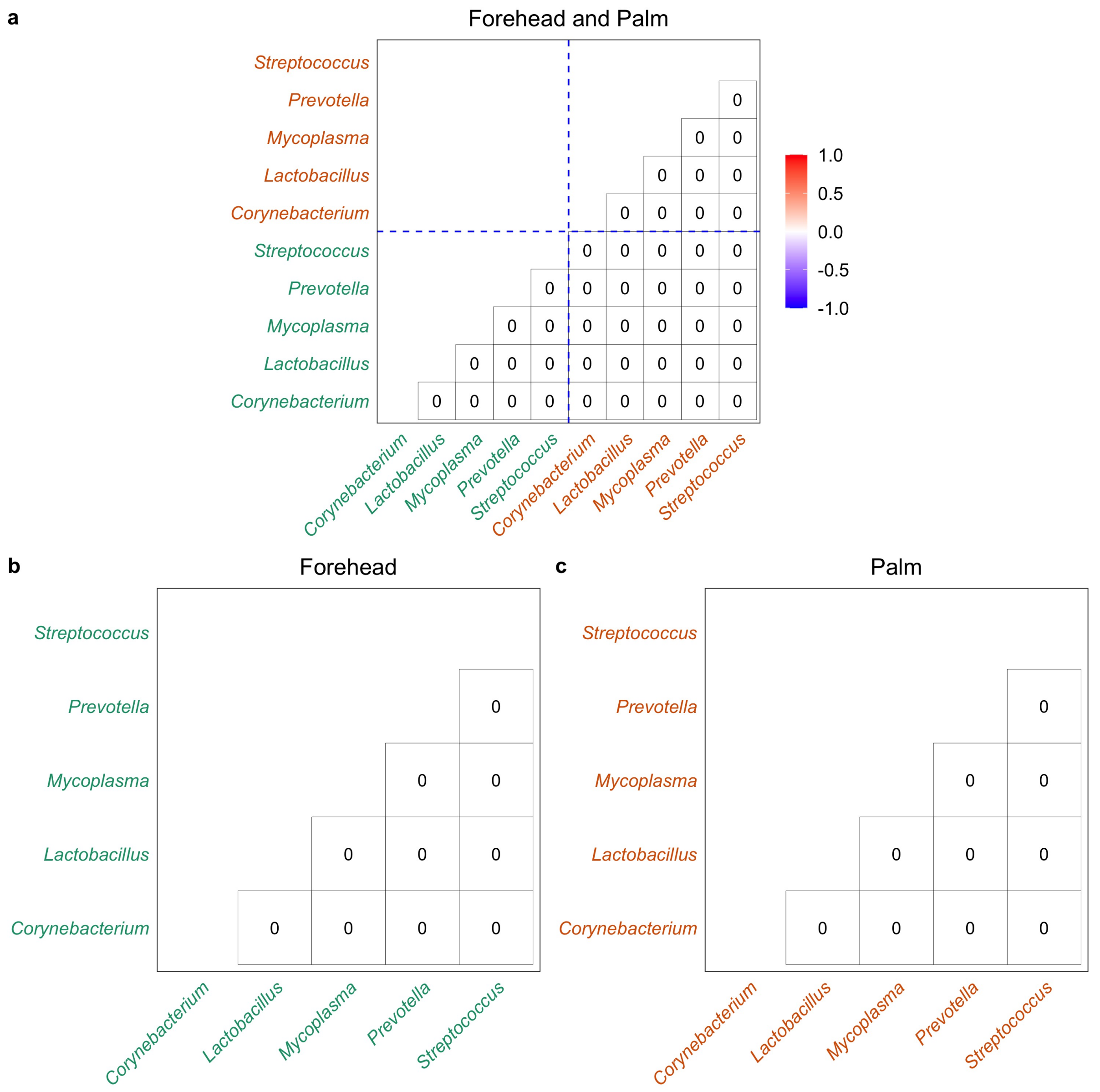

Supplementary Figure 7: Correlations between forehead and palm genera [5] using proportionality method. **a** the correlation matrix calculated by concatenating forehead and palm data, **b** the correlation matrix calculated using the forehead data solely, **c** the correlation matrix calculated using the palm data solely. The top 5 most abundant common genera were selected for the visualization purpose. Genera from forehead are colored in green and genera from palm are colored in brown in x and y axes. The correlation coefficient ranges from -1 to 1, color coded by blue to red, respectively.

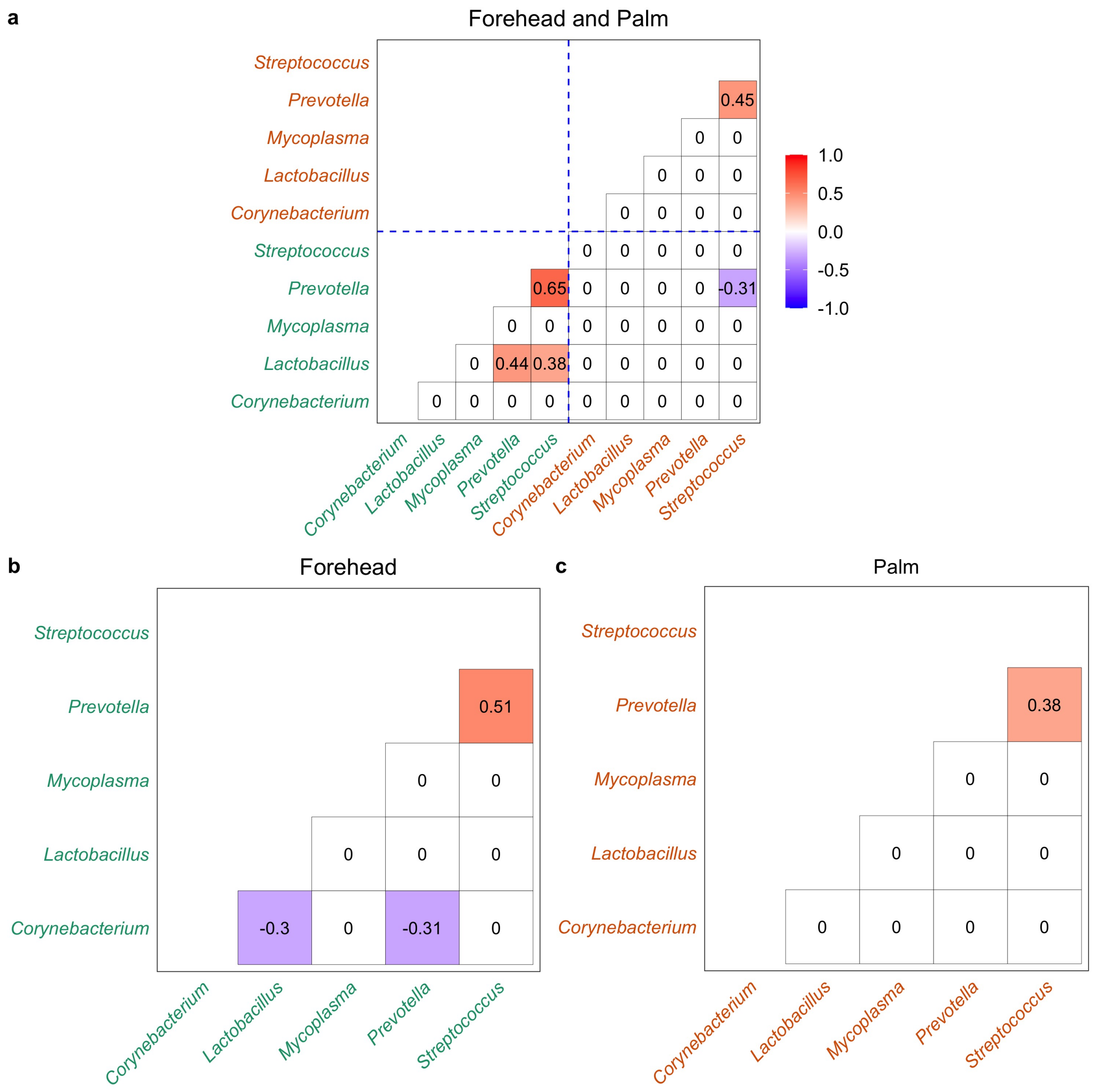

Supplementary Figure 8: Correlations between forehead and palm genera [5] using SparCC. **a** the correlation matrix calculated by concatenating forehead and palm data, **b** the correlation matrix calculated using the forehead data solely, **c** the correlation matrix calculated using the palm data solely. The top 5 most abundant common genera were selected for the visualization purpose. Genera from forehead are colored in green and genera from palm are colored in brown in x and y axes. The correlation coefficient ranges from -1 to 1, color coded by blue to red, respectively.

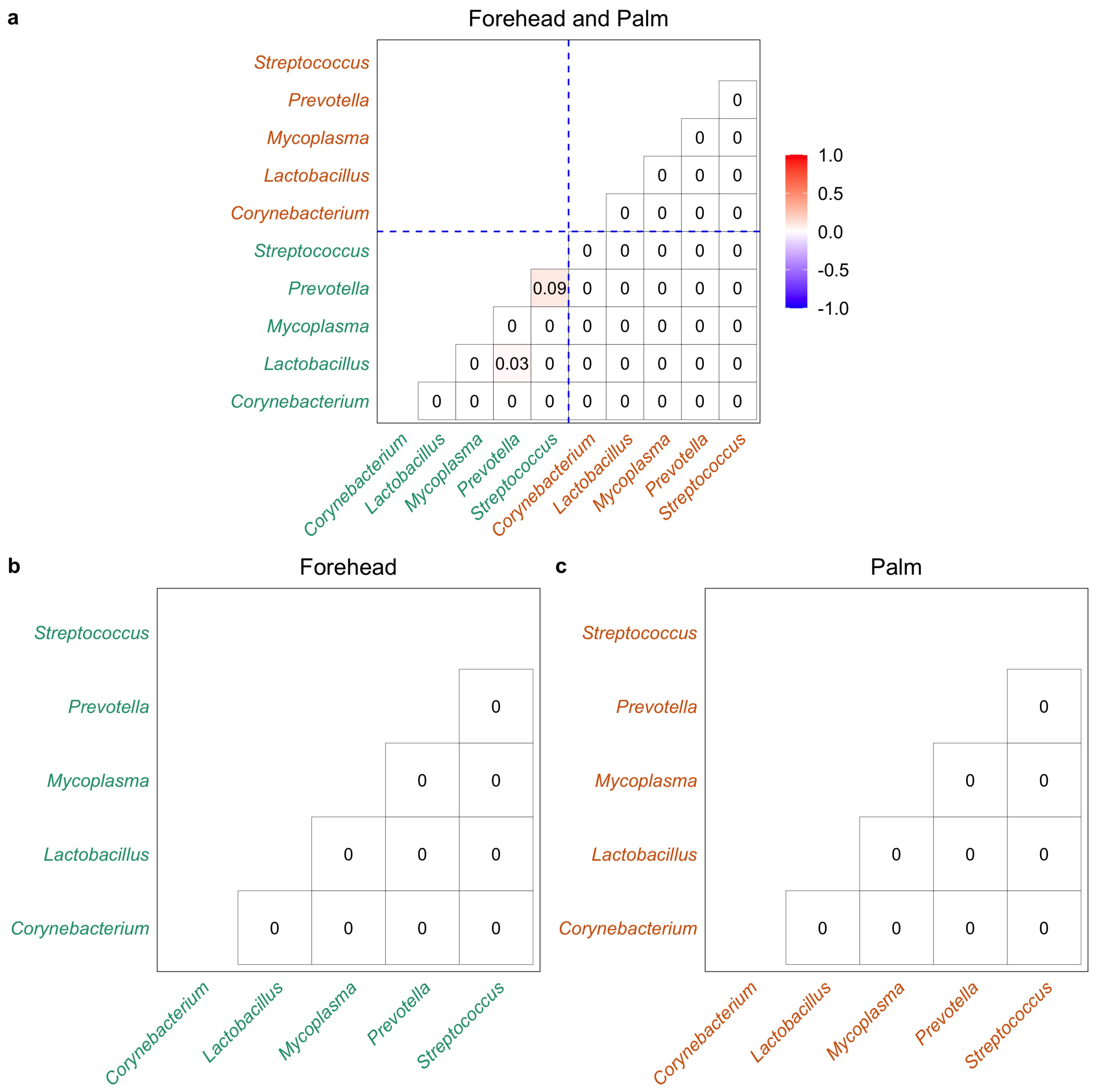

Supplementary Figure 9: Correlations between forehead and palm genera [5] using SPIEC-EASI (MB). **a** the correlation matrices calculated by concatenating forehead and palm data, **b** the correlation matrix calculated using the forehead data solely, **c** the correlation matrix calculated using the palm data solely. The top 5 most abundant common genera were selected for the visualization purpose. Genera from forehead are colored in green and genera from palm are colored in brown in x and y axes. The correlation coefficient ranges from -1 to 1, color coded by blue to red, respectively.

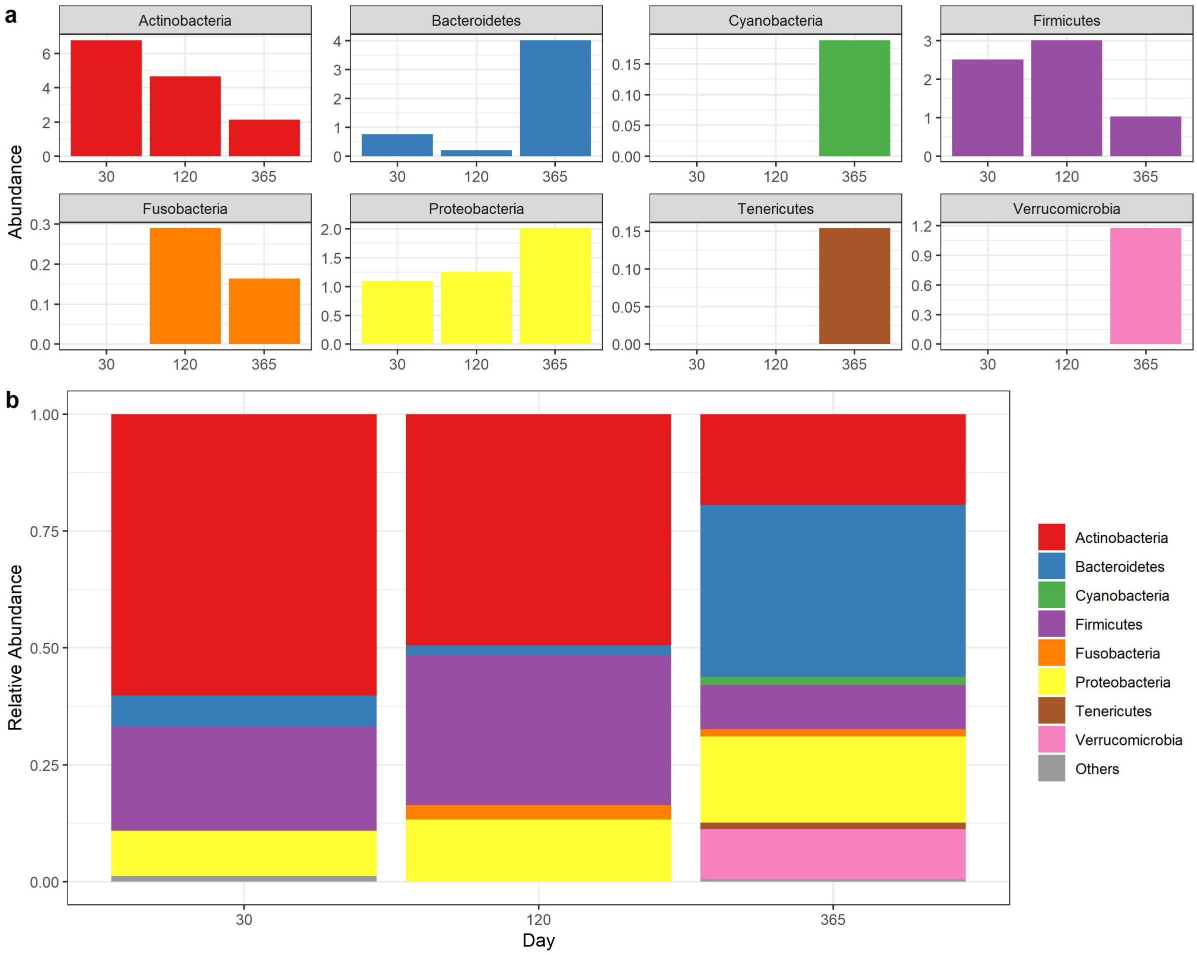

Supplementary Figure 10: Temporal patterns of bias-corrected **a** abundance and **b** relative abundances for Norwegian infant gut microbiome data among phyla. The x-axis represents days (30, 120, and 365 days) and y-axis denotes either the bias-corrected abundances or relative abundances. The top 10 most abundant phyla up to 365 days were selected for the visualization purpose. Phyla with prevalence across samples $< 10\%$ were not shown separately and grouped into "Others". Different taxa were coded by different colors as shown on the legends.

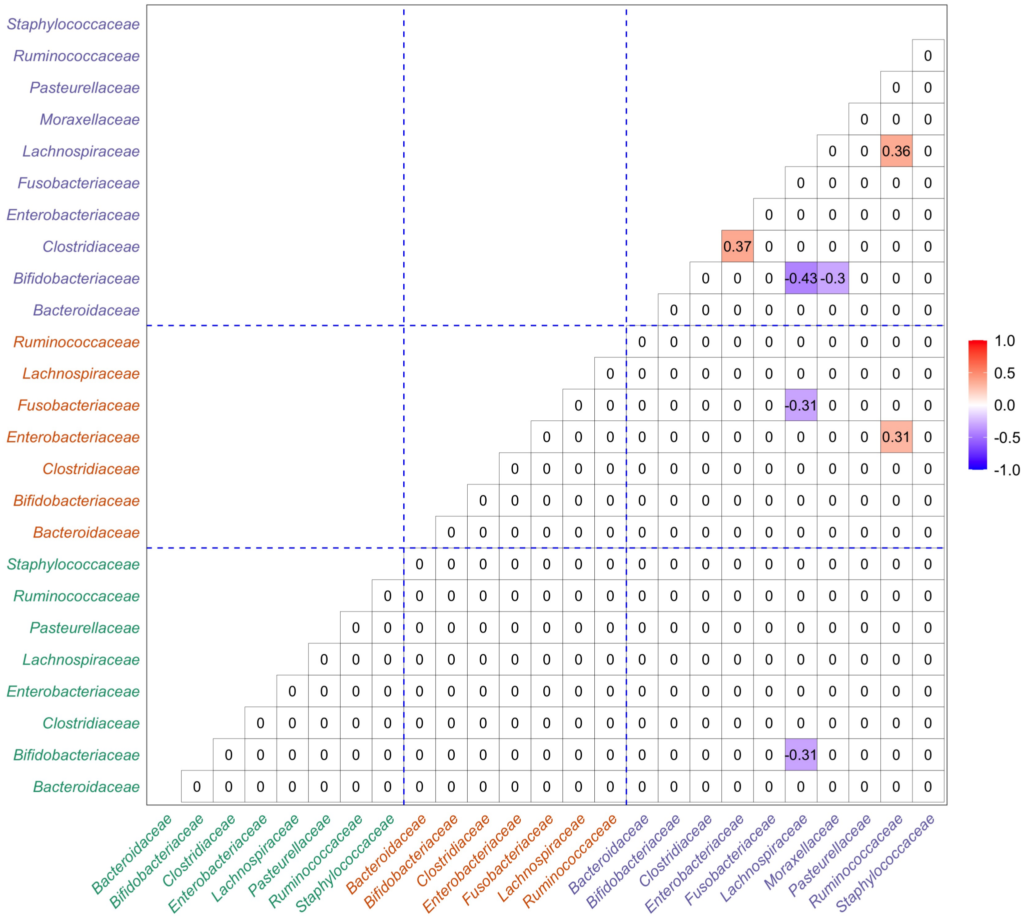

Supplementary Figure 11: Temporal patterns of correlations for Norwegian infant gut microbiome data among families using SparCC. Families from day 30, 120, and 365 are colored in green, brown, and purple in x and y axes, respectively. Blue dashed lines are used to separate families between different time points. The top 10 most abundant families up to 365 days were selected for the visualization purpose. The threshold for SparCC estimates was set at 0.30. Any estimate below 0.3 in its absolute value is coded as 0. The correlation coefficient ranges from -1 to 1, color coded by blue to red, respectively.

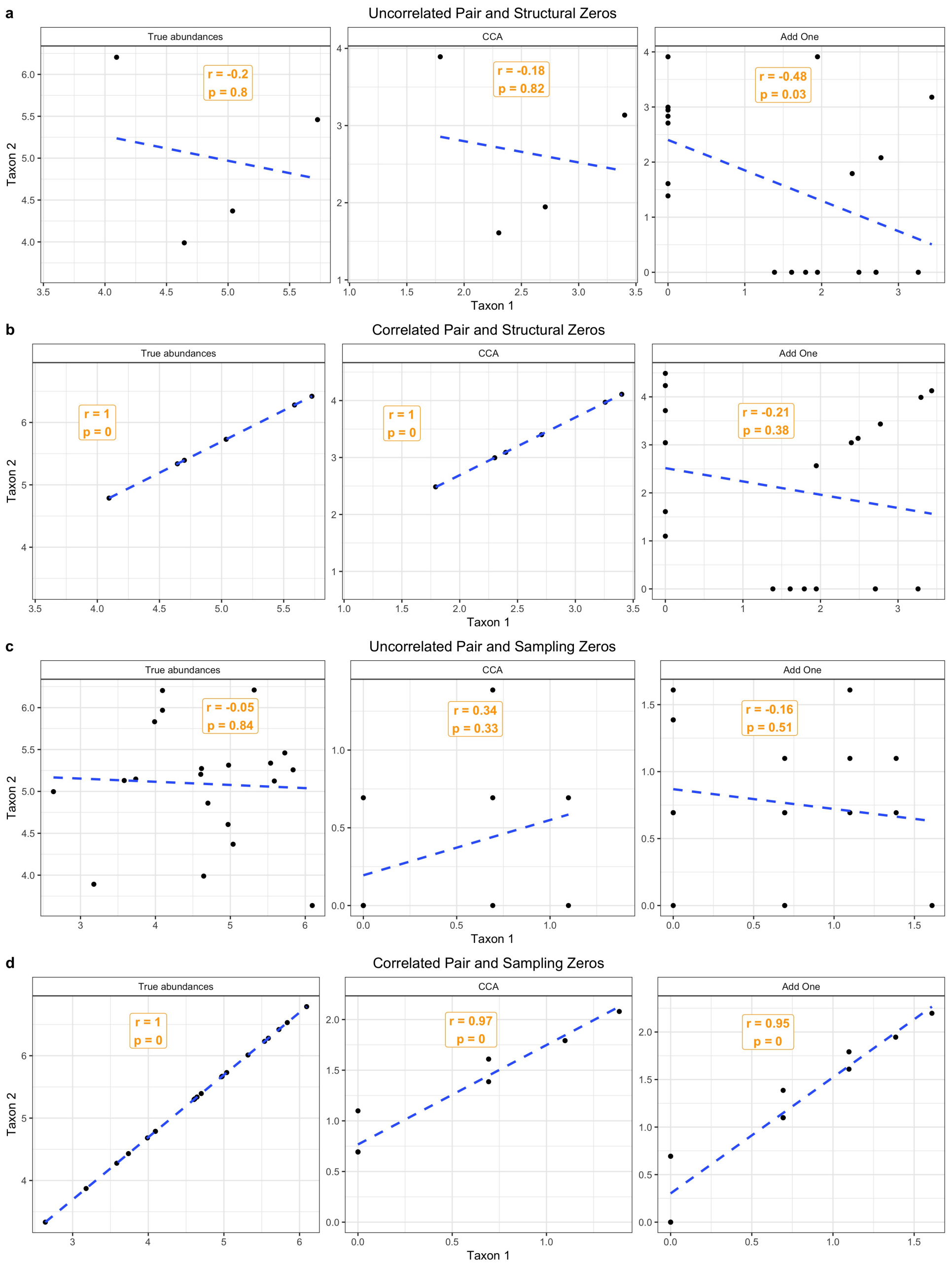

Supplementary Figure 12: Comparisons between complete case analysis (CCA) and adding pseudo-count of one (Add One) in handling structural zeros for **a** uncorrelated taxa and **b** correlated taxa, and sampling zeros for **c** uncorrelated taxa and **d** correlated taxa respectively. Scatter plots are ordered in columns, where the left column represents the true log abundances; the middle column represents the complete (non-zero) log abundances; and the right columns represent the log abundances after adding the pseudo-count of one. The blue dashed line in the plot represents the linear regression fit. The sample Pearson correlation coefficient together with its p-value (two-sided, calculated based on t-distribution, not corrected for multiple comparisons) are shown on the embedded text of each plot.

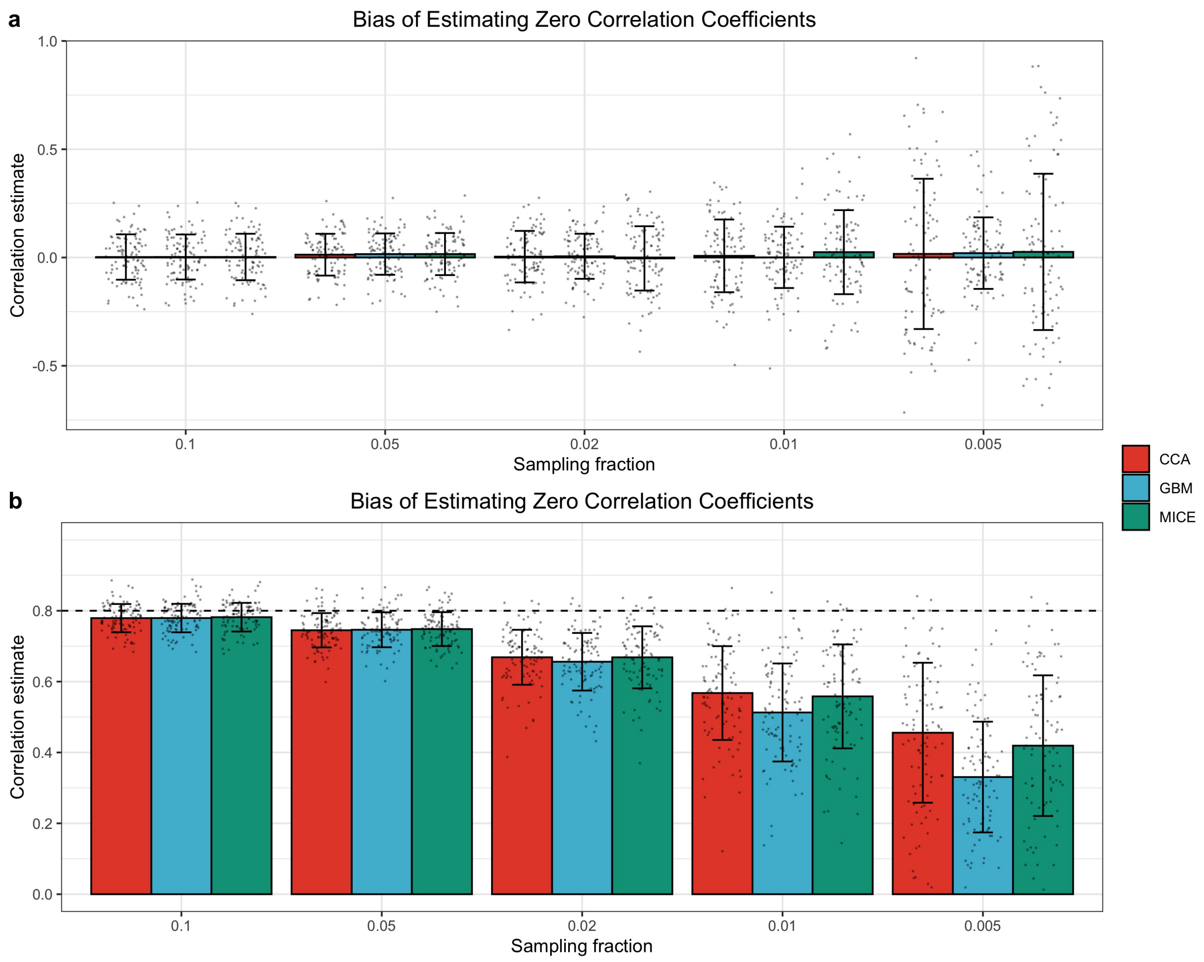

Supplementary Figure 13: Comparisons between complete case analysis (CCA), gradient boosting machine (GBM), and multivariate imputation by chained equations (MICE) in handling zeros for **a** uncorrelated taxa and **b** correlated taxa, respectively. X-axis represents the sampling fractions ranging from 0.1 to 0.005, which corresponds to the proportion of zeros ranging from 0 to around 70%. Y-axis represents the estimated Pearson correlation coefficients. Results are represented by the average of estimated Pearson correlation coefficients ± standard errors (shown as error bars) across 100 simulation runs with the number of samples $n = 100$, the number of taxa $d = 50$, dispersion parameter $\alpha = 2$, and the corresponding sampling fraction specified in the X-axis. Data points are added to the bar charts using dots with jittering. The horizontal dashed line in **b** standards for the true correlation coefficient of 0.8. CCA, GBM, and MICE are colored in red, blue, and green, respectively.

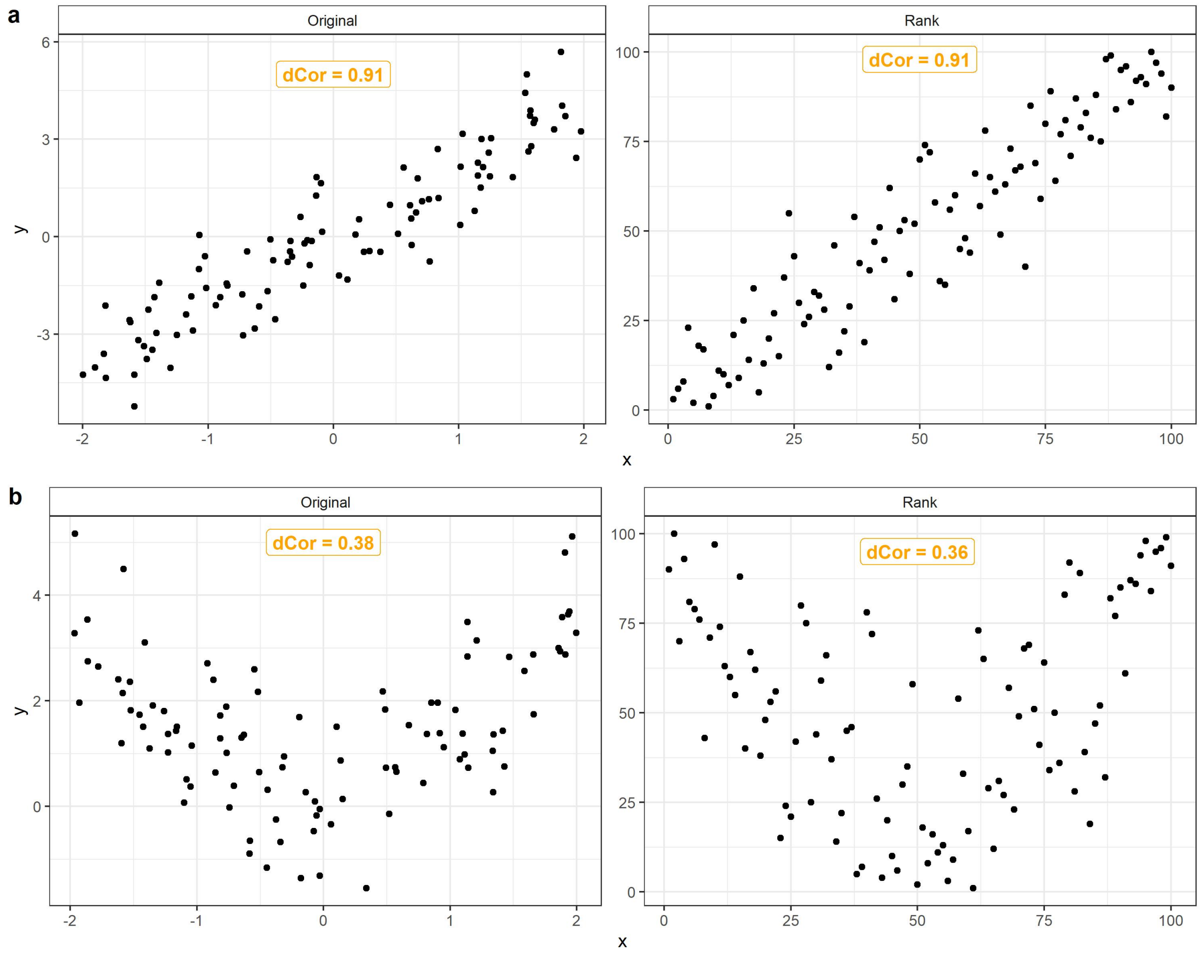

Supplementary Figure 14: Distance correlations calculated using the original data vs. rank-transformed data for **a** linear relationships, **b** nonlinear relationships.

Supplementary Tables

SECOM	Proportionality	SparCC	SPIEC-EASI (MB)	SPIEC-EASI (GL)	Pearson
3.7	1.2	16.1	217.4	357.9	0.2

Supplementary Table 1: Comparison of CPU times (in seconds)

		SECOM (Distance)	
		True	False
SECOM (Pearson2)	True	27.61 (TP = 24.93, FP = 2.68)	3.60 (TP = 0, FP = 3.6)
	False	3.65 (TP = 0.07, FP = 3.58)	4915.14 (TN = 4915.14, FN = 0)

Supplementary Table 2: A 2×2 contingency table of SECOM results using Pearson correlation and distance correlation measures for identifying linear relationships. Simulation setting: $n = 50, d = 100, \alpha = 0.5$, iteration number = 100. Rows represent the results obtained from SECOM (Pearson2) and columns represent the results obtained from SECOM (Distance). Each cell in the table represents a mutually exclusive combination of SECOM (Pearson2) and SECOM (Distance) results (on average of 100 iterations) with true/false positive (TP/FP) or true/false negative (TN/FN) frequencies shown in the parenthesis.

		SECOM (Distance)	
		True	False
SECOM (Pearson2)	True	2.27 (TP = 0, FP = 2.27)	3.95 (TP = 0, FP = 3.95)
	False	25.56 (TP = 22.8, FP = 2.76)	4918.22 (TN = 4916.02, FN = 2.2)

Supplementary Table 3: A 2×2 contingency table of SECOM results using Pearson correlation and distance correlation measures for identifying nonlinear relationships. Simulation setting: $n = 50, d = 100, \alpha = 0.5$, iteration number = 100. Rows represent the results obtained from SECOM (Pearson2) and columns represent the results obtained from SECOM (Distance). Each cell in the table represents a mutually exclusive combination of SECOM (Pearson2) and SECOM (Distance) results (on average of 100 iterations) with true/false positive (TP/FP) or true/false negative (TN/FN) frequencies shown in the parenthesis.

	**N=89**
**Age**	
min	18.00
max	55.00
mean (sd)	23.36 (5.79)
**BMI**	
min	16.82
max	33.84
mean (sd)	22.92 (3.11)
**Gender (%)**	
F	55 (65)
M	30 (35)
NA	4 (4)
**Ethnicity (%)**	
African American	2 (3)
Asian/Pacific island	2 (3)
Caucasian	62 (78)
Caucasian/Asian	1 (1)
Caucasian/Hispanic	1 (1)
Hispanic	7 (9)
Others	2 (2)
NA	10 (11)

Supplementary Table 4: Demographic summary of forehead and palm samples

Family	Day 30	Day 120	Day 365
Bacteroidaceae	0.33	0.14	0.91
Bifidobacteriaceae	0.96	1	1
Clostridiaceae	0.24	0.34	1
Enterobacteriaceae	0.78	0.82	1
Fusobacteriaceae	0.09	0.16	0.15
Lachnospiraceae	0.11	0.3	1
Moraxellaceae	0.02	0	0.15
Pasteurellaceae	0.2	0.02	0.44
Ruminococcaceae	0.59	0.50	0.97
Staphylococcaceae	0.15	0	0.38

Supplementary Table 5: Prevalence of families in NoMIC data

Supplementary References

[1] Jonathan Friedman and Eric J Alm. Inferring correlation networks from genomic survey data. *PLOS computational biology*, 8:e1002687, 2012.

[2] Abhishek Kaul, Ori Davidov, and Shyamal D Peddada. Structural zeros in high-dimensional data with applications to microbiome studies. *Biostatistics*, 18(3):422–433, 2017.

[3] Roman Vershynin. *High-dimensional probability: An introduction with applications in data science*, volume 47. Cambridge university press, 2018.

[4] Gábor J Székely, Maria L Rizzo, and Nail K Bakirov. Measuring and testing dependence by correlation of distances. *The annals of statistics*, 35(6):2769–2794, 2007.

[5] Gilberto E Flores, J Gregory Caporaso, Jessica B Henley, Jai Ram Rideout, Daniel Domogala, John Chase, Jonathan W Leff, Yoshiki Vázquez-Baeza, Antonio Gonzalez, Rob Knight, et al. Temporal variability is a personalized feature of the human microbiome. *Genome biology*, 15(12):1–13, 2014.
