# Supplementary material for: Linear and nonlinear correlation estimators unveil undescribed taxa interactions in microbiome data
Source: Nat Commun. 2022 Aug 23;13:4946. doi: 10.1038/s41467-022-32243-x (PMC9399263; doi:10.1038/s41467-022-32243-x)
Supplement: Supplementary file 3 — Reporting Summary [file 41467_2022_32243_MOESM3_ESM.pdf]

nature portfolio

Corresponding author(s): Shyamal Das Peddada

Last updated by author(s): Jul 6, 2022

Reporting Summary

Nature Portfolio wishes to improve the reproducibility of the work that we publish. This form provides structure for consistency and transparency in reporting. For further information on Nature Portfolio policies, see our Editorial Policies and the Editorial Policy Checklist.

Statistics

For all statistical analyses, confirm that the following items are present in the figure legend, table legend, main text, or Methods section.

n/a	Confirmed	
☐	☒	The exact sample size (n) for each experimental group/condition, given as a discrete number and unit of measurement
☐	☒	A statement on whether measurements were taken from distinct samples or whether the same sample was measured repeatedly
☐	☒	The statistical test(s) used AND whether they are one- or two-sided<br>*Only common tests should be described solely by name; describe more complex techniques in the Methods section.*
☒	☐	A description of all covariates tested
☐	☒	A description of any assumptions or corrections, such as tests of normality and adjustment for multiple comparisons
☐	☒	A full description of the statistical parameters including central tendency (e.g. means) or other basic estimates (e.g. regression coefficient) AND variation (e.g. standard deviation) or associated estimates of uncertainty (e.g. confidence intervals)
☐	☒	For null hypothesis testing, the test statistic (e.g. F, t, r) with confidence intervals, effect sizes, degrees of freedom and P value noted<br>*Give P values as exact values whenever suitable.*
☒	☐	For Bayesian analysis, information on the choice of priors and Markov chain Monte Carlo settings
☒	☐	For hierarchical and complex designs, identification of the appropriate level for tests and full reporting of outcomes
☐	☒	Estimates of effect sizes (e.g. Cohen's d, Pearson's r), indicating how they were calculated

Our web collection on statistics for biologists contains articles on many of the points above.

Software and code

Policy information about availability of computer code

Data collection	Data were processed using RStudio (RStudio 2022.02.3+492, R version 4.2.0) with 1 CPU, x86_64-apple-darwin17.0 (64-bit) and macOS Big Sur/Monterey 10.16<br>Simulation data can be found on GitHub at https://github.com/FrederickHuangLin/SECOM-Code-Archive/tree/v1.0.0. The forehead and palm data can be found in Qiita: forehead https://qiita.ucsd.edu/study/description/2150, palm https://qiita.ucsd.edu/study/description/2149. The NoMIC data used in this study are not publicly available but may be obtained by contacting Dr. Merete Eggesbø at Merete.Eggesbo@fhi.no
Data analysis	Analyses were performed using RStudio (RStudio 2022.02.3+492, R version 4.2.0) with 1 CPU, x86_64-apple-darwin17.0 (64-bit) and macOS Big Sur/Monterey 10.16<br>SECOM has been implemented in the R package ANCOMBC, which is available on Bioconductor at https://www.bioconductor.org/packages/release/bioc/html/ANCOMBC.html. All analyses shown in the paper can be found on GitHub at https://github.com/FrederickHuangLin/SECOM-Code-Archive/tree/v1.0.0

For manuscripts utilizing custom algorithms or software that are central to the research but not yet described in published literature, software must be made available to editors and reviewers. We strongly encourage code deposition in a community repository (e.g. GitHub). See the Nature Portfolio guidelines for submitting code & software for further information.

March 2021

Data

Policy information about availability of data

All manuscripts must include a data availability statement. This statement should provide the following information, where applicable:

- Accession codes, unique identifiers, or web links for publicly available datasets
- A description of any restrictions on data availability
- For clinical datasets or third party data, please ensure that the statement adheres to our policy

Simulation data can be found on GitHub at https://github.com/FrederickHuangLin/SECOM-Code-Archive/tree/v1.0.0. The forehead and palm data can be found in Qiita: forehead https://qiita.ucsd.edu/study/description/2150, palm https://qiita.ucsd.edu/study/description/2149. The NoMIC data used in this study are not publicly available but may be obtained by contacting Dr. Merete Eggesbø at Merete.Eggesbo@fhi.no

Field-specific reporting

Please select the one below that is the best fit for your research. If you are not sure, read the appropriate sections before making your selection.

☒ Life sciences ☐ Behavioural & social sciences ☐ Ecological, evolutionary & environmental sciences

For a reference copy of the document with all sections, see nature.com/documents/nr-reporting-summary-flat.pdf

Life sciences study design

All studies must disclose on these points even when the disclosure is negative.

Sample size	We subset the original NoMIC cohort by restricting to children who were vaginally delivered with no perinatal antibiotics exposure and exclusively breastfed during the first four months of life. The forehead and palm data analyzed in the paper is a publicly available, and we limit our analysis to the data obtained at the first visit (baseline) and to subjects who did not use antibiotics.
Data exclusions	Mothers not fluent in Norwegians were not eligible for the main NoMIC cohort. Children who were not vaginally delivered, or with perinatal antibiotics exposure , or were not exclusively breastfed during the first four months of life were excluded
Replication	Not relevant
Randomization	Not relevant as this was an observational longitudinal population-based cohort- study
Blinding	Not relevant

Reporting for specific materials, systems and methods

We require information from authors about some types of materials, experimental systems and methods used in many studies. Here, indicate whether each material, system or method listed is relevant to your study. If you are not sure if a list item applies to your research, read the appropriate section before selecting a response.

Materials & experimental systems

n/a	Involved in the study	
☒	☐	Antibodies
☒	☐	Eukaryotic cell lines
☒	☐	Palaeontology and archaeology
☒	☐	Animals and other organisms
☒	☐	Human research participants
☒	☐	Clinical data
☒	☐	Dual use research of concern

Methods

n/a	Involved in the study	
☒	☐	ChIP-seq
☒	☐	Flow cytometry
☒	☐	MRI-based neuroimaging
